# Supplementary material for: Structure and inhibition mechanism of the catalytic domain of human squalene epoxidase
Source: Nat Commun. 2019 Jan 9;10:97. doi: 10.1038/s41467-018-07928-x (PMC6327030; doi:10.1038/s41467-018-07928-x)
Supplement: Supplementary file 1 — Supplementary Information [file 41467_2018_7928_MOESM1_ESM.pdf]

Supplementary Information

Structure and inhibition mechanism of the catalytic domain of human squalene epoxidase

A. K. Padyana et al.

a

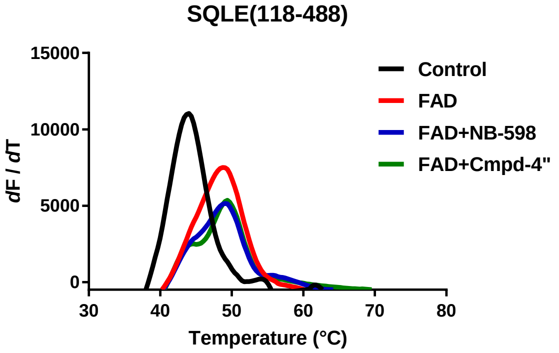

b

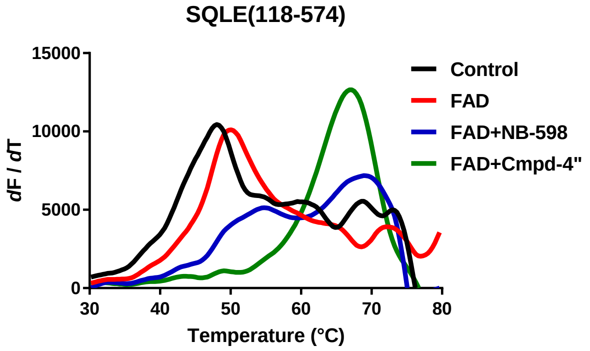

c

Compounds	SQLE(118-488)		SQLE(118-574)	
	mean T_m (°C)	ΔT_m (°C)	mean T_m (°C)	ΔT_m (°C)
Control	44.0	0	48.0	0
FAD	48.7	4.7	50.1	2.1
FAD+NB-598	49.1	5.1	68.9	19.9
FAD+Cmpd-4”	49.4	5.4	67.2	17.2

Supplementary Figure 1 | Thermal shift assay results and construct selection for crystallography studies.

(**a**) Thermal shift assay fluorescence signals obtained for SQLE (118-488), with and without FAD, and the addition of NB-598 and Cmpd-4’’ inhibitors. The first derivative response of fluorescence (*d*F/*d*T) is plotted against the temperature.

(**b**) Thermal shift assay fluorescence signals obtained for SQLE (118-574), with and without FAD, and the addition of NB-598 and Cmpd-4’’ inhibitors. The first derivative response of fluorescence (*d*F/*d*T) is plotted against the temperature.

(**c**) Table listing the calculated T_m and shift in T_m (ΔT_m) values for SQLE (118-488) and SQLE (118-574) derived from (**a**) and (**b**). SQLE constructs lacking C-terminus (118-488) showed a significant shift for FAD that suggests stabilization upon binding, but failed to show further stabilization upon addition of the inhibitors. In contrast, the SQLE (118-574) construct that included C-terminal membrane-binding domain showed more modest stabilization after FAD addition, but displayed pronounced stabilization upon addition of NB-598 and Cmpd-4’’ inhibitors. Results obtained here suggest that while a soluble domain (118-488) is sufficient for FAD interaction, the C-terminal segment 489-574 is required for inhibitor binding, leading to selection of SQLE (118-574) for structural studies.

a

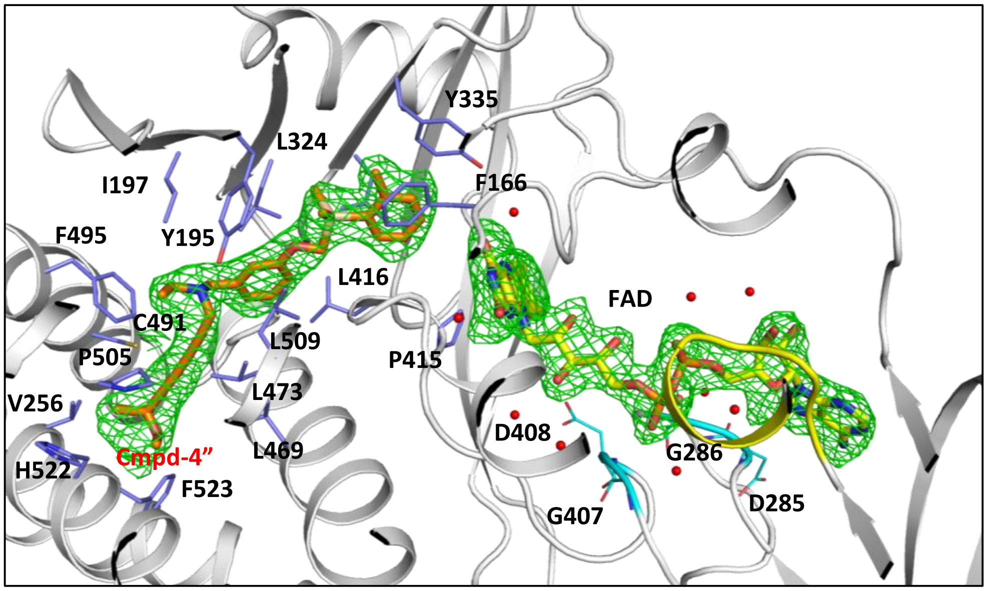

b

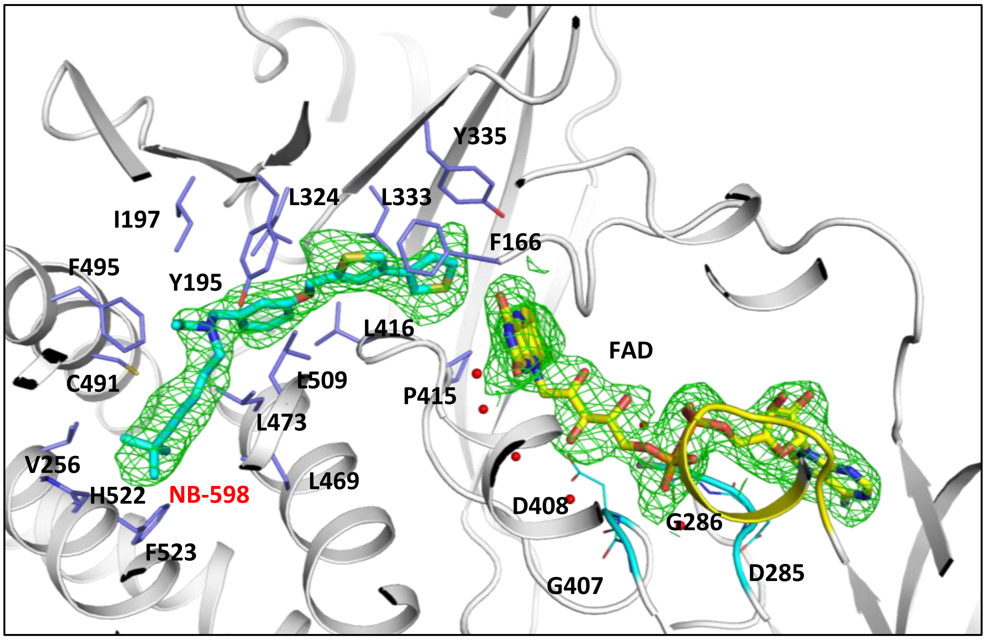

Supplementary Figure 2 | Electron density for FAD and inhibitor at SQLE active site. SQLE structure is in cartoon presentation in grey. Water molecules are presented as red spheres. The residues in the Cmpd-4” and NB-598 binding site are presented as thin sticks and labeled. F_o - F_c electron density omit map, calculated by simulated annealing procedure and contoured at 3.0 σ surrounding FAD and the inhibitors (Cmpd-4” or NB-598 shown as sticks), is presented as a green mesh. The conserved motifs for FAD recognition are shown in yellow (GXGXXG) and cyan (DG/GD).

(**a**) Cmpd-4” bound structure (SQLE•FAD•Cmpd-4”).

(**b**) NB-598 bound structure (SQLE•FAD•NB-598).

Supplementary Figure 3

a

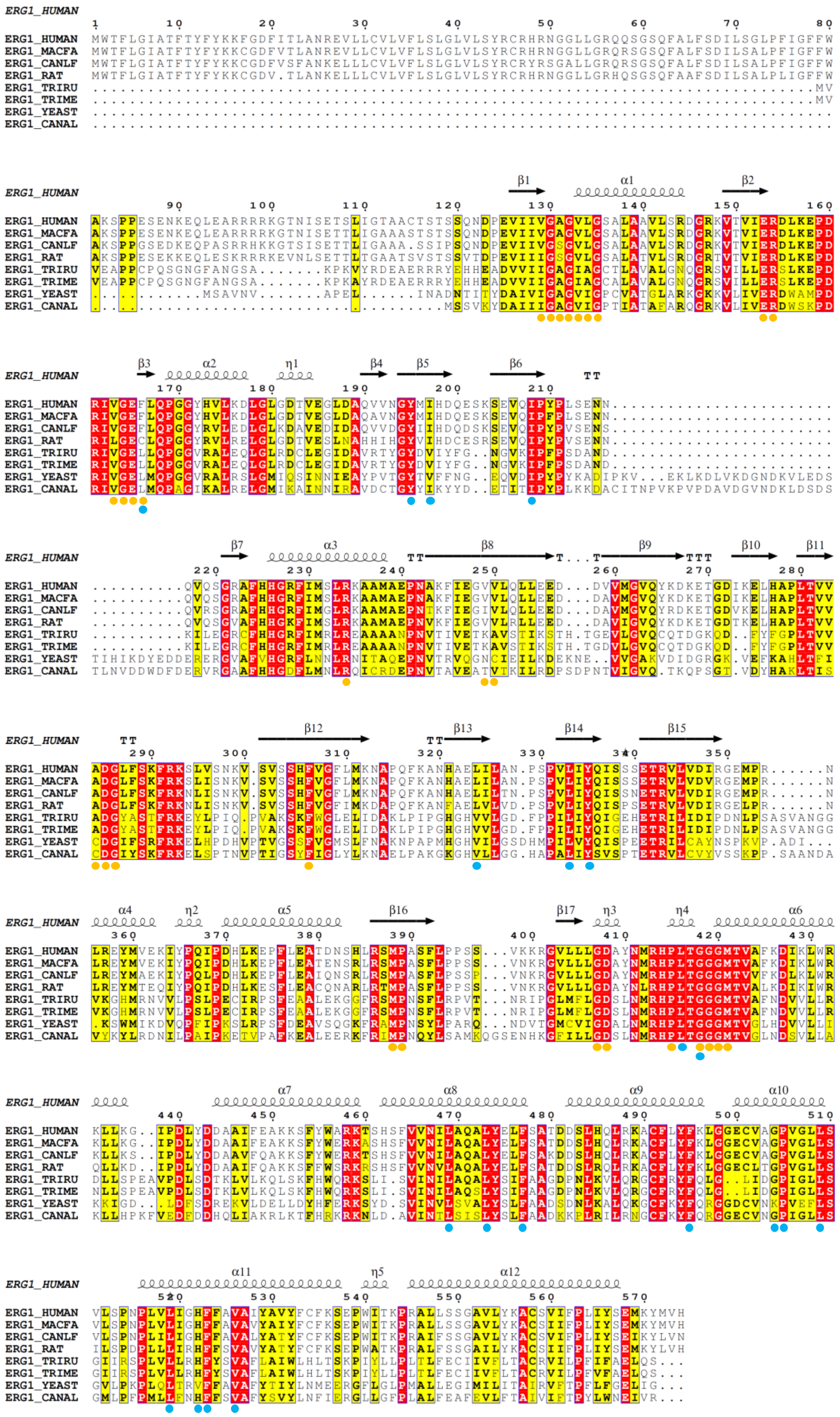

b

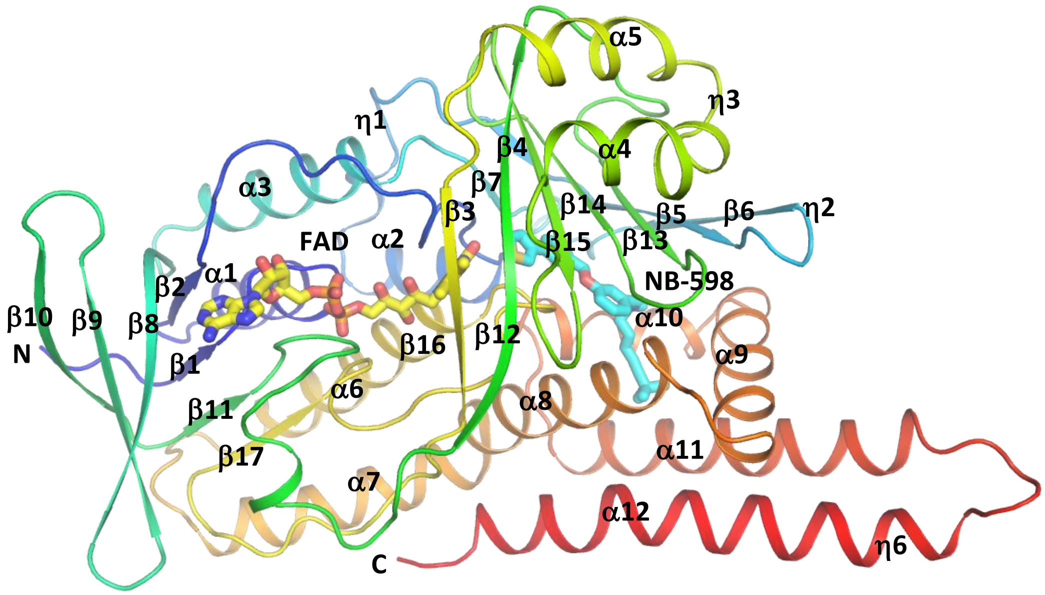

Supplementary Figure 3 | Sequence conservation, secondary and tertiary structures of SQLE bound to inhibitor NB-598.

(**a**) Sequence alignment of select mammalian and fungal SQLE orthologs along with secondary structure annotations derived from the SQLE•FAD•NB-598 tertiary structure (panel **b**) is shown above the aligned sequences. Eight sequences are displayed corresponding to UniProt database entries ERG1_HUMAN (Q14534) *Homo Sapiens*, ERG1_CYNO (G7PCT2) *Macaca fascicularis*, ERG1_DOG (E2RHB9) *Canis lupus familiaris,* ERG1_RAT (P52020) *Rattus norvegicus,* ERG1_TRIRU (Q4JEX9) *Trichophyton rubrum*, ERG1_TRIME (A0A1U9IDN6) *Trichophyton mentagrophytes,* ERG1_YEAST (P32476) *Saccharomyces cerevisiae (strain ATCC 204508 / S288c)* and ERG1_CANAL (Q92206) *Candida albicans (strain SC5314 / ATCC MYA-2876)*. Conserved amino acids that are identical between the aligned sequences are colored in red, similar residues are in yellow and non-conserved in white. Human SQLE amino acids engaged in direct interactions with FAD and NB-598 observed in the crystal structure are annotated below the alignment with orange and blue dots, respectively.

(**b**) The tertiary structure of SQLE•FAD•NB-598 is shown with ribbon representation using a rainbow color ramp of violet to red variation from N- to C-term. FAD and NB-598 are in stick representation with the carbon atoms in yellow and cyan, respectively. Secondary structural elements consisting α-helices (α), β-strands (β) and 3_{10}-turns and helices (η) are annotated on the tertiary structure.

a

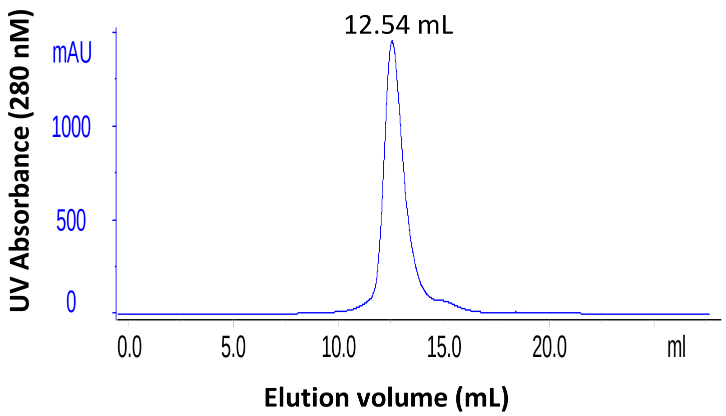

b

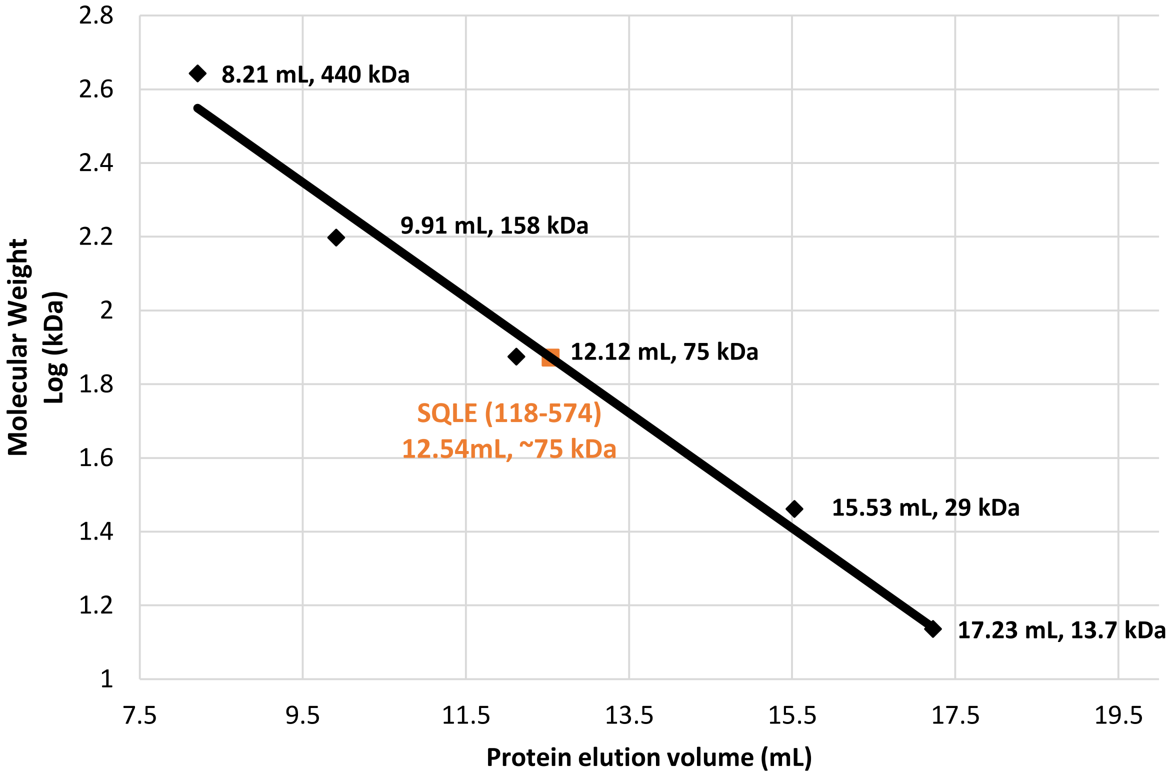

Supplementary Figure 4 | Size-exclusion chromatography analysis of SQLE (118-574) protein.

(**a**) SEC elution profile for SQLE (118-574) on Superdex 200 Increase 10/300 Column.
(**b**) The estimated molecular weight (MW) for SQLE (118-574) determined based on the MW standards eluted using the same Superdex 200 Increase 10/300 column. The black diamonds represent MW standards: horse spleen Ferritin - 440 kDa, rabbit muscle Aldolase - 158 kDa, chicken egg white Conalbumin - 75 kDa, bovine erythrocytes Carbonic anhydrase - 29 kDa, bovine pancreas Ribonucleose A - 13.7 kDa (Gel Filtration Calibration kit LMW and HMW, GE Healthcare) with their elution volume in mL along with MW labels adjacent to their position on the graph. SQLE (118-574) elution volume in mL was plotted on the standard curve and indicated as an orange square with estimated MW derived from the plot (~75kDa, orange text). The calculated MW for SQLE (118-574) is 50.8 kDa.

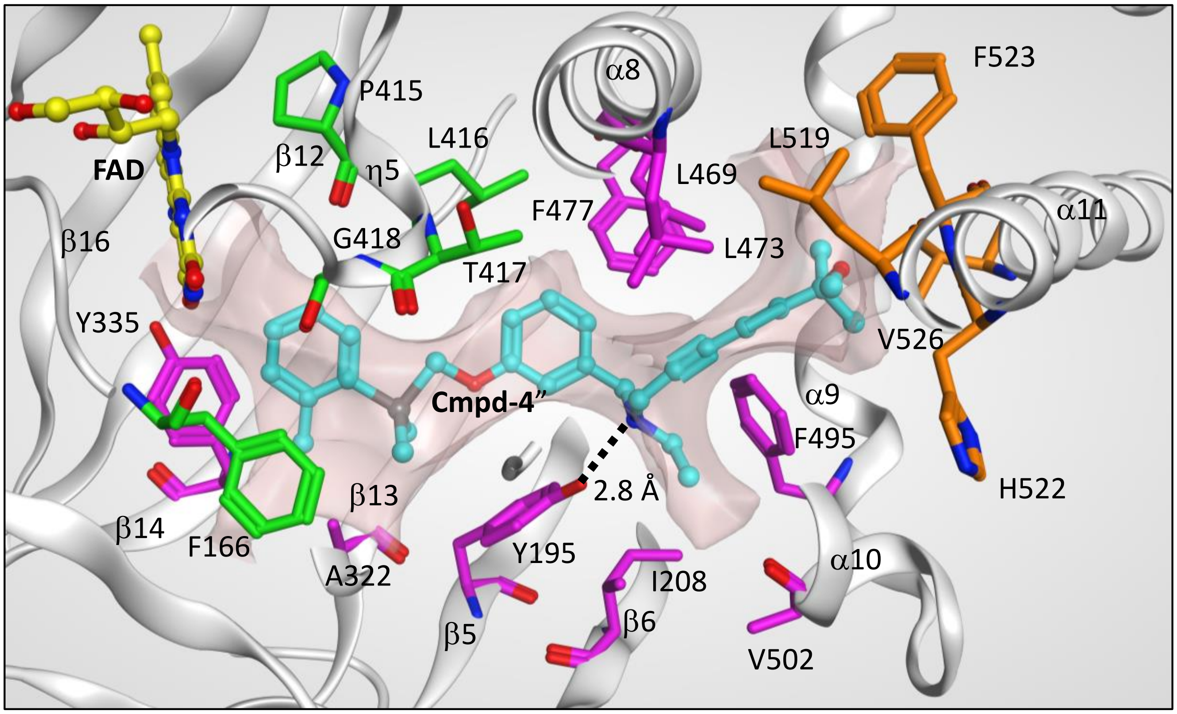

Supplementary Figure 5 | Cmpd-4" binding site. Cmpd-4" is in ball-and-stick representation with carbon atoms in cyan and the VDW interaction contact surface of the binding pocket within the 4.5Å of Cmpd-4" shown in semi-translucent surface. FAD is in ball-and-stick presentation with carbon atoms in yellow. The residues forming the compound binding site are in line representation with the color scheme to match the domain coloring scheme as described before with important specific residues shown as sticks. Hydrogen bond interaction between Y195 and the central amine of Cmpd-4" is shown as a dotted line with the distance labeled.

a

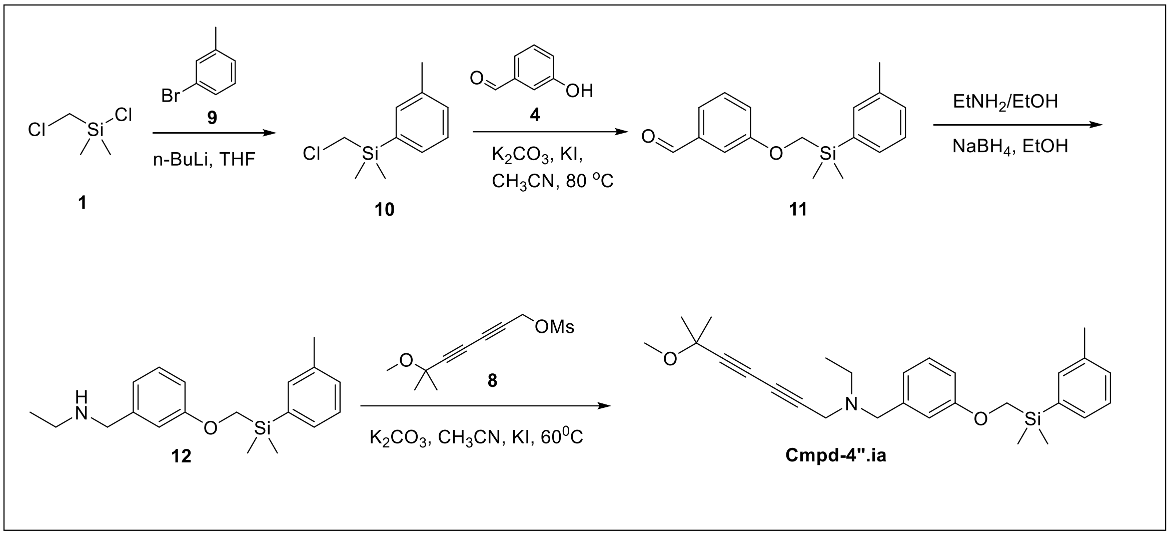

b

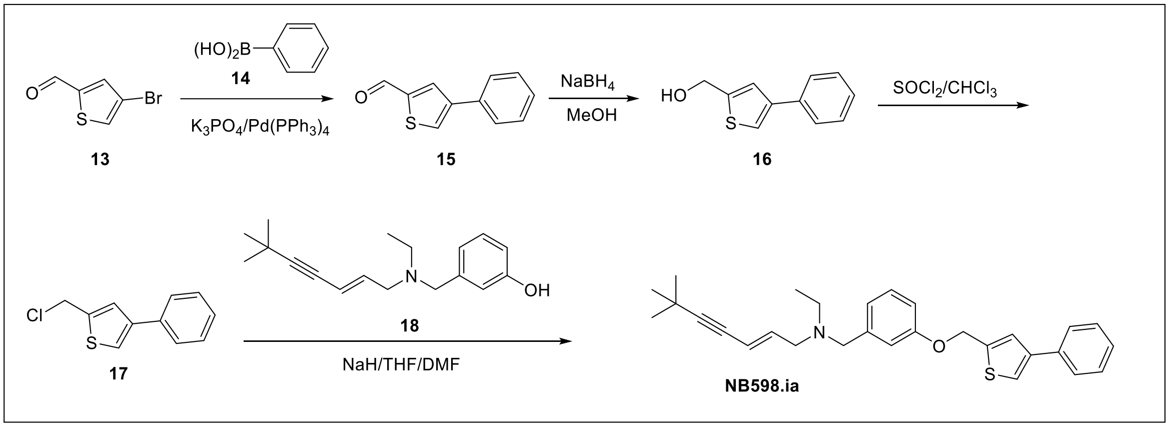

Supplementary Figure 6 | Scheme for chemical synthesis of inactive inhibitor analogs.
(**a**) Four step synthesis scheme for the inactive analog of Cmpd-4” (**Cmpd-4”.ia,** see **Supplementary Methods).**
(**b**) Four step synthesis scheme for the inactive analog of NB-598 (**NB-598.ia**, see **Supplementary Methods).**

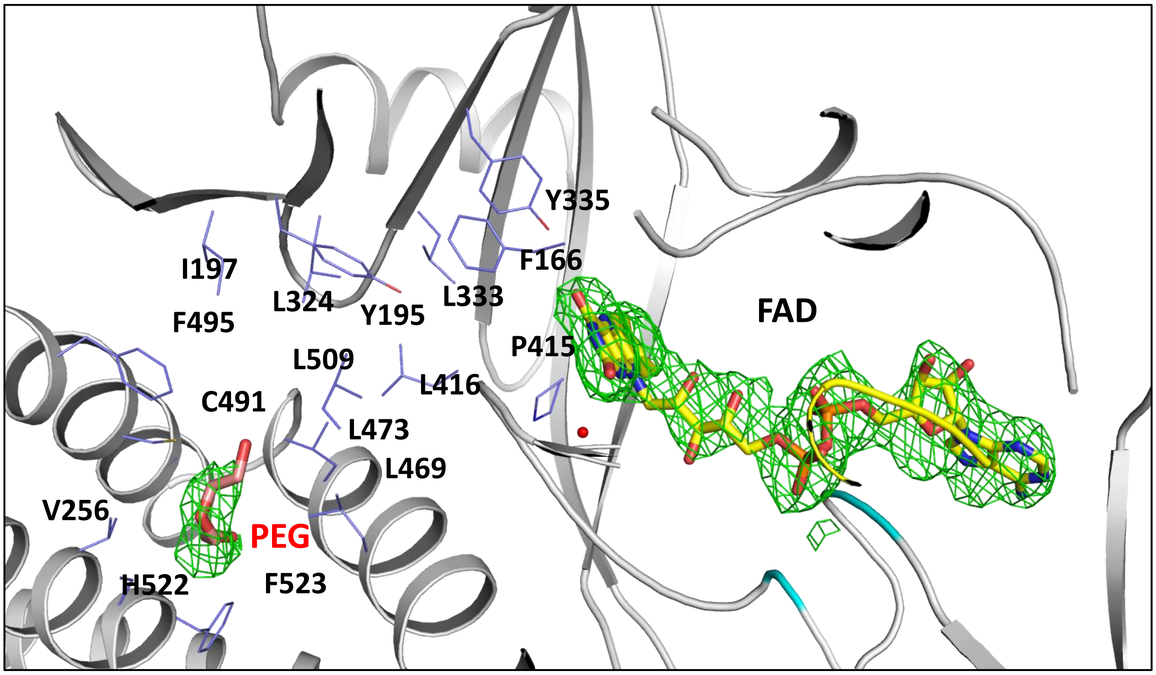

Supplementary Figure 7 | Electron density at the active site of unliganded SQLE structure. SQLE structure is in grey cartoon representation. Water molecules are presented as red spheres. Equivalent residues from the inhibitor binding sites (**Supplementary Fig. 2)** are represented as thin sticks and labeled. F_o - F_c electron density omit maps calculated by simulated annealing procedure and contoured at 3.0 σ surrounding FAD and the polyethylene glycol molecule (PEG, shown as sticks) are presented as a green mesh. The conserved motifs for FAD recognition are shown in yellow (GXGXXG) and cyan (DG/GD).

Supplementary Table 1. Point mutation in fungal SQLE reported to be resistant to Terbinafine inhibition. Equivalent amino acid residues in human SQLE were mapped from the sequence alignment.

Organism	Mutation	Human equivalent	Reference
Trichophyton rubrum	F397L	F477	Osborne et al., 2005[1]
	L393F	L473	Osborne et al., 2006[2]
	L393F/S	L473	Yamada et al., 2017[3]
	F397L/I/V	F477	
	F415I/S/V	F492	
	H440Y	H522	
Saccharomyces cerevisiae	P430A	P505	Klobucnikova *et al.*, 2003[4]
	L251F	L326	Leber et al., 2003[5]
	F402L	F477	
	F420L	F495	
	P430S	P505	
	F433S	L508	

Supplementary Table 2. Primer sequence for SQLE plasmids.

Plasmid Name	F/R*	Primer Sequence (5' to 3')
pET28a-N-His-MBP-TEV-SQLE (1-488)	F	GAAAACCTGTATTTTCAGGGCATGTGGACTTTTCTGGGCA
	R	GCTTTGTTAGCAGCCGGATCTCATCATCTTAGTTGATGCAGGGAA
pET28a-N-His-MBP-TEV-SQLE (1-518)	F	GAAAACCTGTATTTTCAGGGCATGTGGACTTTTCTGGGCA
	R	GCTTTGTTAGCAGCCGGATCTCATCAAACTAGAGGGTTAGGAGACA
pET28a-N-His-MBP-TEV-SQLE (1-543)	F	GAAAACCTGTATTTTCAGGGCATGTGGACTTTTCTGGGCA
	R	GCTTTGTTAGCAGCCGGATCTCATTTTGTAATCCAAGGTTCTGACTTAAA
pET28a-N-His-MBP-TEV-SQLE (1-574)	F	GAAAACCTGTATTTTCAGGGCATGTGGACTTTTCTGGGCA
	R	GCTTTGTTAGCAGCCGGATCTCATCACTTATCGTCGTCAT
pET28a-N-His-MBP-TEV-SQLE (101-488)	F	GAAAACCTGTATTTTCAGGGCGGAACCAATATTTCAGAAACAAG
	R	GCTTTGTTAGCAGCCGGATCTCATCATCTTAGTTGATGCAGGGAA
pET28a-N-His-MBP-TEV-SQLE (101-518)	F	GAAAACCTGTATTTTCAGGGCGGAACCAATATTTCAGAAACAAG
	R	GCTTTGTTAGCAGCCGGATCTCATCAAACTAGAGGGTTAGGAGACA
pET28a-N-His-MBP-TEV-SQLE (101-543)	F	GAAAACCTGTATTTTCAGGGCGGAACCAATATTTCAGAAACAAG
	R	GCTTTGTTAGCAGCCGGATCTCATTTTGTAATCCAAGGTTCTGACTTAAA
pET28a-N-His-MBP-TEV-SQLE (101-574)	F	GAAAACCTGTATTTTCAGGGCGGAACCAATATTTCAGAAACAAG
	R	GCTTTGTTAGCAGCCGGATCTCATCACTTATCGTCGTCAT
pET28a-N-His-MBP-TEV-SQLE (111-488)	F	GAAAACCTGTATTTTCAGGGCGGAACAGCTGCCTGTACATCAACA
	R	GCTTTGTTAGCAGCCGGATCTCATCATCTTAGTTGATGCAGGGAA
pET28a-N-His-MBP-TEV-SQLE (111-518)	F	GAAAACCTGTATTTTCAGGGCGGAACAGCTGCCTGTACATCAACA
	R	GCTTTGTTAGCAGCCGGATCTCATCAAACTAGAGGGTTAGGAGACA
pET28a-N-His-MBP-TEV-SQLE (111-543)	F	GAAAACCTGTATTTTCAGGGCGGAACAGCTGCCTGTACATCAACA
	R	GCTTTGTTAGCAGCCGGATCTCA TTTTGTAATCCAAGGTTCTGACTTAAA
pET28a-N-His-MBP-TEV-hSQLE (111-574)	F	GAAAACCTGTATTTTCAGGGCGGAACAGCTGCCTGTACATCAACA
	R	GCTTTGTTAGCAGCCGGATCTCATCACTTATCGTCGTCAT
pET28a-N-His-MBP-TEV-SQLE (118-488)	F	GAAAACCTGTATTTTCAGGGCACATCTTCTCAGAATGACCCAGAA
	R	GCTTTGTTAGCAGCCGGATCTCATCATCTTAGTTGATGCAGGGAA
pET28a-N-His-MBP-TEV-SQLE (118-518)	F	GAAAACCTGTATTTTCAGGGCACATCTTCTCAGAATGACCCAGAA
	R	GCTTTGTTAGCAGCCGGATCTCATCAAACTAGAGGGTTAGGAGACA
pET28a-N-His-MBP-TEV-SQLE (118-543)	F	GAAAACCTGTATTTTCAGGGCACATCTTCTCAGAATGACCCAGAA
	R	GCTTTGTTAGCAGCCGGATCTCATTTTGTAATCCAAGGTTCTGACTTAAA
pET28a-N-His-MBP-TEV-SQLE (118-574)	F	GAAAACCTGTATTTTCAGGGCACATCTTCTCAGAATGACCCAGAA
	R	GCTTTGTTAGCAGCCGGATCTCATCACTTATCGTCGTCAT
pET28a-N-His-MBP-TEV-SQLE (124-488)	F	GAAAACCTGTATTTTCAGGGCCCAGAAGTTATCATCGTGGGAGCT
	R	GCTTTGTTAGCAGCCGGATCTCATCATCTTAGTTGATGCAGGGAA
pET28a-N-His-MBP-TEV-SQLE (124-518)	F	GAAAACCTGTATTTTCAGGGC CCAGAAGTTATCATCGTGGGAGCT
	R	GCTTTGTTAGCAGCCGGATCTCATCAAACTAGAGGGTTAGGAGACA
pET28a-N-His-MBP-TEV-SQLE (124-543)	F	GAAAACCTGTATTTTCAGGGCCCAGAAGTTATCATCGTGGGAGCT
	R	GCTTTGTTAGCAGCCGGATCTCATTTTGTAATCCAAGGTTCTGACTTAAA
pET28a-N-His-MBP-TEV-SQLE (124-574)	F	GAAAACCTGTATTTTCAGGGCCCAGAAGTTATCATCGTGGGAGCT
	R	GCTTTGTTAGCAGCCGGATCTCATCACTTATCGTCGTCAT

**Plasmid Name**	**F/R***	**Primer Sequence (5' to 3')**
pET28a-N-His-MBP-TEV-SQLE (144-488)	F	GAAAACCTGTATTTTCAGGGCAGAGATGGAAGAAAGGTGACA
	R	GCTTTGTTAGCAGCCGGATCTCATCATCTTAGTTGATGCAGGGAA
pET28a-N-His-MBP-TEV-SQLE (144-518)	F	GAAAACCTGTATTTTCAGGGCAGAGATGGAAGAAAGGTGACA
	R	GCTTTGTTAGCAGCCGGATCTCATCAAACTAGAGGGTTAGGAGACA
pET28a-N-His-MBP-TEV-SQLE (144-543)	F	GAAAACCTGTATTTTCAGGGCAGAGATGGAAGAAAGGTGACA
	R	GCTTTGTTAGCAGCCGGATCTCATTTTGTAATCCAAGGTTCTGACTTAAA
pET28a-N-His-MBP-TEV-SQLE (144-574)	F	GAAAACCTGTATTTTCAGGGCAGAGATGGAAGAAAGGTGACA
	R	GCTTTGTTAGCAGCCGGATCTCATCACTTATCGTCGTCAT

***Forward (F) or Reverse (R)

Supplementary Methods

General synthetic procedures and experimental notes.

In the following examples, the chemical reagents were purchased from commercial sources (such as Alfa, Acros, Sigma Aldrich, TCI and Shanghai Chemical Reagent Company) and used without further purification. Flash chromatography was performed on an Isolera One column (Biotage) with silica gel particles of 200–300 mesh. Analytical and preparative thin-layer chromatography (prep-TLC) plates were HSGF 254 (0.15–0.2 mm thickness, Shanghai Anbang Company). Nuclear magnetic resonance (NMR) spectra were recorded using an AMX-400 NMR and Avance II 600MHz spectrometers (Brucker). Chemical shifts were reported in parts per million (ppm, δ) downfield from tetramethylsilane. Mass spectra were run with electrospray ionization (ESI) from an LCT TOF mass spectrometer (Waters). HPLC chromatographs were recorded on a LC-20AHT (Shimadzu) liquid chromatography system (column: Ultimate 4.6 mm × 50 mm [Welch], 5µm, mobile phase A:H_2O/MeCN/TFA=90/10/0.1; mobile phase B: MeCN/H_2O/TFA=90/10/0.1). NB-598 (96.0% pure, HPLC) and Cmpd-4" (99.7% pure, HPLC) were prepared following published experimental procedures[6, 7]**.** Following abbreviations are used in describing the synthesis methods - THF: tetrahydrofuran, MBTE: tert-butyl methyl ether, PE: petroleum ether, r.t.: room temperature, DCM: dichloromethane, TEA: triethyl amine, DMF: dimethylformamide, TFA: trifluoroacetic acid.

Synthesis of 10 [(chloromethyl)dimethyl(m-tolyl)silane]

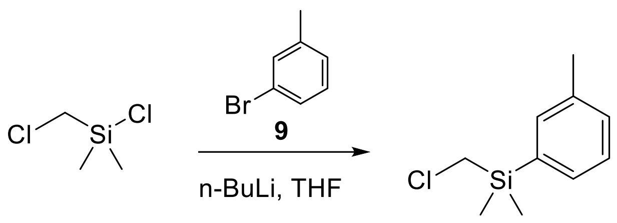

1

10

To a stirred mixture of 1-bromo-3-methylbenzene **(9)** (1.00 g, 5.88 mmol) in THF (10 mL) cooled to -78 °C, was added dropwise *n*-BuLi (2.5M in hexane, 2.35 mL). The reaction mixture was stirred at -78 °C for 0.5 h, then **1** (918 mg, 6.4 mmol) was added dropwise. The resulting mixture was allowed to warm to 0 °C and then stirred for 1h. The solution was quenched with aqueous NH_4Cl solution and extracted with EtOAc twice. The organic phase was washed by brine, dried over anhydrous Na_2SO_4, and concentrated under reduced pressure. The residue was filtered through a pad of silica gel (PE: EtOAc= 30:1) to give the crude product **10** (1.00 g, yield: 86.0%) as a light oil, which was used directly for next step reaction.

Synthesis of 11 [3-((dimethyl(m-tolyl)silyl)methoxy)benzaldehyde

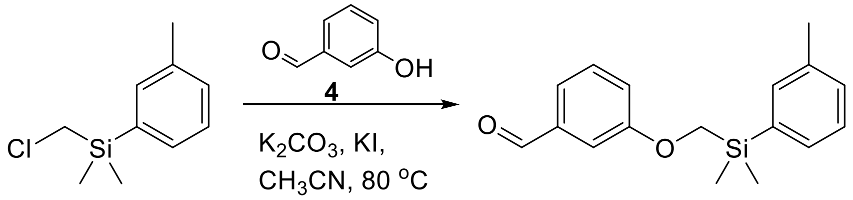

10

11

To a stirred mixture of **4** (739 mg, 6.0 mmol), K_2CO_3 (1.40 g, 10.1 mmol) and KI (284 mg, 2.0 mmol) in CH_3CN (10 mL), was added **10** (1.00 g, 5.05 mmol) at r.t. The reaction mixture was stirred at 80 °C overnight. The mixture was then cooled to r.t and filtered. The filtrate was concentrated, and the

residue was purified by prep-TLC (PE: EtOAc=3:1) to give the desired product **11** (500 mg, 36.0%) as a light oil, LCMS: m/z 285 $(M+H)^+$

Synthesis of 12 [N-(3-((dimethyl(m-tolyl)silyl)methoxy)benzyl)ethanamine]

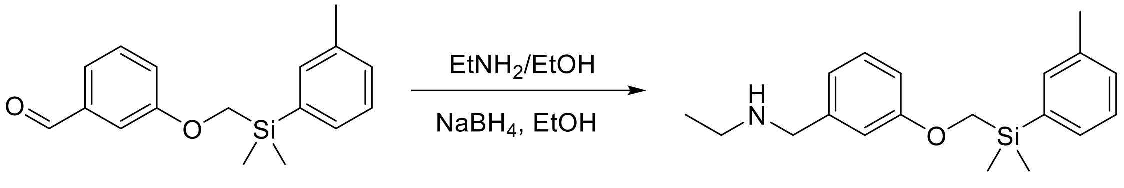

11 **12**

A mixture of **11** (500 mg, 1.7 mmol) in 30% $EtNH_2$/EtOH (5 mL) solution and 3A molecular sieves (1 g) was stirred at r.t. overnight. $NaBH_4$ (76 mg, 3.4 mmol) was then added portion-wise to the solution at 0 ^oC. After the addition, the reaction mixture was stirred at r.t for 1 h. The reaction mixture was filtered, and the filtrate was concentrated under reduced pressure. The residue was purified by silica gel chromatography (DCM: MeOH=20: 1) to give the product **12** (300 mg, yield: 56.3%) as a white solid. LCMS: m/z 314 $(M+H)^+$

^1H NMR (400 MHz, $CDCl_3$) δ 7.32 (d, *J* = 4.0 Hz, 2H), 7.22 – 7.16 (m, 2H), 7.15 – 7.02 (m, 3H), 6.86 (dd, *J* = 8.0, 2.1 Hz, 1H), 3.90 (s, 2H), 3.72 (s, 2H), 2.76 (q, *J* = 7.3 Hz, 2H), 2.29 (s, 3H), 1.30 (t, *J* = 8.0 Hz, 3H), 0.35 (s, 6H).

Synthesis of Cmpd-4".ia [N-(3-((dimethyl(m-tolyl)silyl)methoxy)benzyl)-N-ethyl-6-methoxy-6-methylhepta-2,4-diyn-1-amine]

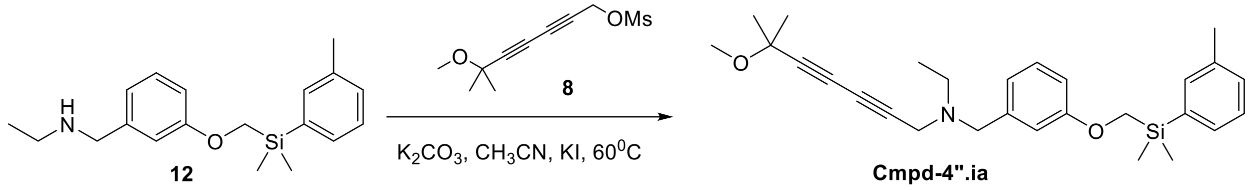

To a stirred mixture of **12** (60 mg, 0.19 mmol), K_2CO_3 (52 mg, 0.38 mmol) and KI (10 mg) in CH_3CN (5 mL) was added **8** (119 mg, 0.52 mmol) at r.t. The reaction mixture was stirred at 60 °C for 2 h. The reaction mixture was cooled to r.t and then filtered. The filtrate was concentrated under reduced pressure. The residue was purified by prep-TLC (PE: EtOAc= 2:1) to give desired product **Cmpd-4".ia** (40.6 mg, yield: 48.3%) as a white solid, 99.6% pure (HPLC). LCMS: m/z 448 $(M+H)^+$

^{1}H NMR (400 MHz, $CDCl_3$) δ 7.35 – 7.30 (m, 2H), 7.22-7.11 (m,, 3H),, 6.91 (s, 1H), 6.85 – 6.76 (m, 2H), 3.69 (s, 2H), 3.53 (s, 2H), 3.35 (s, 2H), 3.30 (s, 3H), 2.55 (q, *J* = 7.1 Hz, 2H), 2.29 (s, 3H), 1.40 (s, 6H), 1.04 (t, *J* = 7.1 Hz, 3H), 0.34 (d, *J* = 3.3 Hz, 6H). ^{13}C NMR (151 MHz, DMSO) δ 161.45, 140.50, 137.17, 136.77, 134.76, 131.29, 130.54, 129.62, 128.23, 121.08, 114.70, 113.28, 79.86, 77.20, 70.90, 68.97, 68.65, 60.36, 57.82, 51.74, 47.12, 41.73, 28.20, 21.57, 12.98, -3.99.

Synthesis of 15 [4-phenylthiophene-2-carbaldehyde]

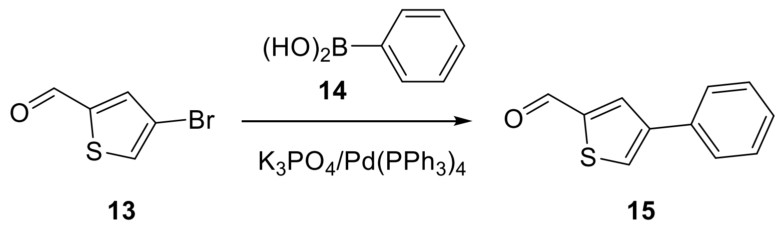

To a stirred solution of 4-bromothiophene-2-carbaldehyde **(13)** (1.50 g, 7.85 mmol), phenylboronic acid **(14)** (1.05 mmol, 8.63 mmol) and $Pd(PPh_3)_4$, (540 mg, 0.47 mmol) in DMF (20 mL), was added K_3PO_4 (4.99 g, 23.5 mmol) at r.t. The reaction mixture was stirred at 80 °C under N_2 atmosphere overnight. The

mixture was cooled to r.t, diluted with H_2O, and extracted with EtOAc twice. The organic phase was washed by brine, dried over anhydrous Na_2SO_4, and concentrated under reduced pressure. The residue was purified by silica gel chromatography (PE: EtOAc= 3:1) to give the desired product **15** (1.10 g, yield: 75.8%) as a white solid. LCMS: m/z 189 $(M+H)^+$

Synthesis of 16 [(4-phenylthiophen-2-yl)methanol]

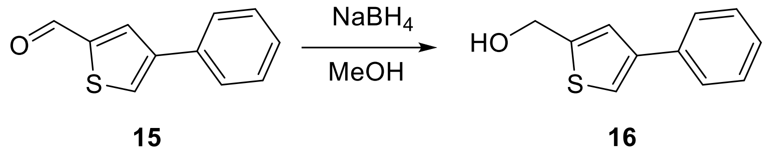

To a stirred solution of **15** (1.10 g, 5.85 mmol) in MeOH (15 mL), $NaBH_4$ (333 mg, 8.77 mmol) was added portion-wise at 0 ^oC over a period of 5 min. The reaction mixture was stirred at r.t for 20 min. The mixture was concentrated, and the residue was diluted with H_2O, and extracted with DCM. The organic phase was washed by brine, dried over anhydrous Na_2SO_4, and concentrated under reduced pressure to give the crude product **16** (1.10 g, yield: 100%) as a white solid. LCMS: m/z 191 $(M+H)^+$

Synthesis of 17 [2-(chloromethyl)-4-phenylthiophene]

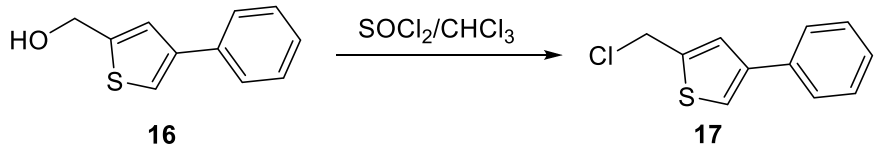

To a stirred mixture of **16** (200 mg, 1.05 mmol) in $CHCl_3$ (10 mL) and 1 drop DMF, $SOCl_2$ (0.2 mL) was added at 0 ^oC. The reaction mixture was stirred at r.t for 15 min and concentrated under vacuo. The residue was diluted with DCM, washed with saturated aqueous $NaHCO_3$ solution. The resulting mixture

was extracted with DCM. The organic phase was washed with brine, dried over anhydrous Na_2SO_4 and concentrated under reduced pressure to give the crude product **17** which was used directly in the next step.

Synthesis of NB598.ia [(E)-N-ethyl-6,6-dimethyl-N-(3-((4-phenylthiophen-2-yl)methoxy)benzyl)hept-2-en-4-yn-1-amine]

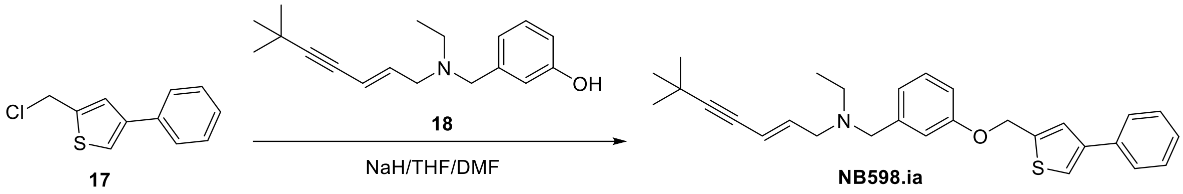

Compound **18** was prepared following procedure as described[7]. To a solution of **18** (100 mg, 0.36 mmol) in THF (5 mL) was added NaH (200 mg, 60% in mineral oil) at 0 °C. The reaction mixture was stirred at 0 °C for 5 minutes. Then a solution of crude **17** (200 mg, 1.05 mmol) in DMF (2 mL) was added. The resulting mixture was stirred at r.t overnight. The mixture was poured into water and extracted with EtOAc twice. The organic phase was washed by brine, dried over anhydrous Na_2SO_4, and concentrated under reduced pressure. The residue was purified by prep-HPLC to give the desired product **NB598.ia** (38.6 mg, yield: 24.2%) as a sticky oil, 99.9% pure (HPLC). LCMS: m/z 444 $(M+H)^+$

^{1}H NMR (400 MHz, $CDCl_3$) δ 7.5 (d, *J* = 1.2Hz, 2H), 7.39 – 7.28 (m, 4H), 7.24 – 7.19 (m, 3H), 6.96 – 6.87 (m, 2H), 6.05-6.00 (m, 1H), 5.66 (d, *J* = 15.6 Hz, 1H), 5.21 (s, 2H), 3.77 (s, 2H), 3.30 (d, *J* = 7.2Hz, 2H), 2.75 – 2.66 (m, 2H), 1.24 – 1.04 (m, 12H). ^{13}C NMR (150 MHz, CDCl3) δ 158.38, 142.04, 141.60, 140.29, 139.66, 135.76, 129.20, 128.82, 127.21, 126.35, 126.04, 121.75, 120.88, 115.17, 113.42, 112.27, 98.24, 77.29, 65.13, 57.60, 55.13, 47.26, 31.05, 27.91, 12.03.

Supplementary References

1. Osborne, C. S. et al. Biological, biochemical, and molecular characterization of a new clinical *Trichophyton rubrum* isolate resistant to terbinafine. *Antimicrob. Agents Chemother.* **50**, 2234-2236 (2006).
2. Osborne, C. S., Leitner, I., Favre, B. & Ryder, N. S. Amino acid substitution in *Trichophyton rubrum* squalene epoxidase associated with resistance to terbinafine. *Antimicrob. Agents Chemother.* **49**, 2840-2844 (2005).
3. Yamada, T. et al. Terbinafine resistance of *Trichophyton* clinical isolates caused by specific point mutations in the squalene epoxidase gene. *Antimicrob. Agents Chemother.* **61**, 1-13 (2017).
4. Klobucnikova, V. et al. Terbinafine resistance in a pleiotropic yeast mutant is caused by a single point mutation in the *ERG1* gene. *Biochem. Biophys. Res. Commun.* **309**, 666-671 (2003).
5. Leber, R. et al. Molecular mechanism of terbinafine resistance in *Saccharomyces cerevisiae. Antimicrob. Agents Chemother.* **47**, 3890-3900 (2003).
6. Takezawa, H. et al. Substitued alkylamine derivatives. *US Patent* **5,234,946** (1993).
7. Gotteland, J. P. et al. (Aryloxy)methylsilane derivatives as new cholesterol biosynthesis inhibitors: synthesis and hypocholesterolemic activity of a new class of squalene epoxidase inhibitors. *J. Med. Chem.* **38**, 3207-3216 (1995).
